# Decreased defensive reactivity to interoceptive threat after successful exposure-based psychotherapy in patients with panic disorder

**DOI:** 10.1038/s41398-021-01298-7

**Published:** 2021-03-17

**Authors:** Christoph Benke, Manuela G. Alius, Alfons O. Hamm, Christiane A. Pané-Farré

**Affiliations:** 1 Department of Psychology, Clinical Psychology, Experimental Psychopathology, and Psychotherapy, Philipps University of Marburg, Gutenbergstraße 18, 35032 Marburg, Germany; 2 Department of Physiological and Clinical Psychology/ Psychotherapy, University of Greifswald, Franz-Mehring-Str. 47, 17487 Greifswald, Germany; 3 Center for Mind, Brain and Behavior (CMBB), University of Marburg and University of Giessen, Marburg, Germany

**Keywords:** Psychiatric disorders, Neuroscience

## Abstract

Panic disorder (PD) is characterized by a dysfunctional defensive responding to panic-related body symptoms that is assumed to contribute to the persistence of panic symptomatology. The present study aimed at examining whether this dysfunctional defensive reactivity to panic-related body symptoms would no longer be present following successful cognitive behavior therapy (CBT) but would persist when patients show insufficient symptom improvement. Therefore, in the present study, effects of CBT on reported symptoms and defensive response mobilization during interoceptive challenge were investigated using hyperventilation as a respiratory symptom provocation procedure. Changes in defensive mobilization to body symptoms in the course of CBT were investigated in patients with a primary diagnosis of PD with or without agoraphobia by applying a highly standardized hyperventilation task prior to and after a manual-based CBT (*n* = 38) or a waiting period (wait-list controls: *n* = 20). Defensive activation was indexed by the potentiation of the amygdala-dependent startle eyeblink response. All patients showed a pronounced defensive response mobilization to body symptoms at baseline. After treatment, no startle reflex potentiation was found in those patients who showed a clinically significant improvement. However, wait-list controls and treatment non-responders continued to show increased defensive responses to actually innocuous body symptoms after the treatment/waiting period. The present results indicate that the elimination of defensive reactivity to actually innocuous body symptoms might be a neurobiological correlate and indicator of successful CBT in patients with PD, which may help to monitor and optimize CBT outcomes.

## Introduction

From a mechanistic perspective, anxiety pathologies have been discussed to be associated with dysfunctions in brain systems responsible for defensive responding to threat^1–3^. As such, the central characteristic of anxiety disorders is the initiation of exaggerated defensive mobilization at lower intensity and greater distance of threat^4,5^. Panic disorder (PD), one of the most debilitating anxiety disorders, is characterized by multiple facets of overexpressed defensive mobilization: out of the blue massive defensive alarm states, that is, panic attacks, occurring in the absence of explicit external threat cues. As a consequence of such panic attacks, persistent anxious apprehension often accompanied by maladaptive changes in behavior is triggered by mild body symptoms signaling an upcoming panic attack^6^.

Cognitive behavior therapy (CBT) for PD has been demonstrated to be effective in reducing the described panic pathology^7^. During CBT, patients are encouraged to repeatedly face feared bodily symptoms and situations during interoceptive and in situ exposure exercises. It is assumed that these exercises enable an extinction learning process during which inhibitory networks are strengthened that downregulate excessive responding of subcortical defensive systems^8^. Traditionally, the key criterion to validate successful treatment has been the reduction of patient’s reported symptoms, i.e., the number of panic attacks, the severity of experienced panic symptoms, anxious apprehension as well as dysfunctional avoidance^7^. Thus, the question arises to what extent changes of the response output of these defensive brain networks are associated with treatment success.

Recent etiological models of PD have stressed that panic-related body symptoms bear specific relevance for patients with PD and are implicated in dysfunctional defensive responding in PD^6^. Aiming to capture the dysfunction of defensive systems in panic pathology, previous psychophysiological and brain imaging studies have demonstrated increased defensive responding to experimentally evoked innocuous bodily symptoms, i.e., interoceptive threat in patients with PD or at-risk individuals^9–13^. Using respiratory symptom provocation tasks (e.g., hyperventilation (HV)), interoceptive conditioning paradigms or biological challenges (e.g., administration of caffeine), PD patients, and at-risk individuals were characterized by increased defensive network activation as well as increased defensive reflex responses^9,10,12^, indicating exaggerated defensive mobilization to evoked body symptoms.

Effects of therapeutic interventions on defensive network activation in PD have recently been investigated in standard fear paradigms (e.g., picture viewing, fear conditioning, or threat of shock) using standard threat material (e.g., loud noise, electrical shock, aversive pictures)^14–19^. These studies indicated a normalization of general activation in brain defensive networks in a fear-conditioning paradigm after CBT^15,18^. Interestingly, impaired clinical improvement co-occurred with persistence of dysfunctional activation patterns in defensive brain circuits^17^. In a threat of shock paradigm, patients with anxiety disorders including PD patients showed a decrease of defensive reflexes after CBT^14^. While these data are promising as they indicate a decrease in general defensive reactivity after CBT, previous studies did not account for the pivotal role of dysfunctional defensive reactivity to *feared body symptoms* for the persistence of panic symptomatology. One might assume that the elimination of such dysfunctional defensive reactivity to *feared body symptoms*, which are assumed to be specifically relevant in PD, is a crucial mechanism responsible for the improvement of panic symptomatology during CBT—a hypothesis that has not been tested yet. A better understanding of this potential mechanism of symptom improvement would help to specifically target this mechanism during treatment to improve CBT for PD patients.

The present study, therefore, examined the effects of CBT on defensive response mobilization to feared body symptoms in relation to clinical improvement as assessed with self-report. To establish an interoceptive threat, a guided and highly controlled HV task was applied, which has been demonstrated to reliably provoke panic-relevant symptoms like breathlessness, palpitations, or dizziness^20,21^. It was previously demonstrated that the startle reflex—a low-level brain stem reflex—was increased when high anxiety-sensitive individuals, i.e. healthy persons who parallel patients with PD in their fear of body symptoms^9,22^, are exposed to this task. Research in animals and humans has revealed the modulation of the startle reflex as a cross-species readout of activity of the defensive brain system centered on the amygdala^23–25^. Studies in traumatized or anxiety disorder patients repeatedly demonstrated increased startle reflex response during exposure to perceived threat which has been linked to overexpressed defensive circuit activity in these disorders^26–29^. Moreover, evidence in anxious populations has indicated that the amygdala-dependent modulation of the startle reflex is a sensitive measure to monitor treatment-related changes in defensive circuit activity^14,30,31^. In the present study, the potentiation of the startle reflex was therefore assessed as the primary outcome measure of changes in defensive circuit mobilization in a cohort of CBT-treated PD patients vs. a PD patient wait-list control group. To evaluate the relation of therapeutic success as indicated by verbal report in relation to changes in defensive responding to threat, in line with previous studies^7,17^, the treated PD patients where categorized for analyses as therapy responders (those who reached a—based on subjective report data—pre-defined high functioning level after therapy) vs. non-responders (those who did not reach the pre-defined criterion).

Based on previous studies^9,20,21,32,33^, we assumed that the HV task will lead to an increase in panic-related bodily symptoms, heart rate, skin conductance level, and a compensatory decrease in respiratory rate^9,22^. It has been demonstrated that guided HV repeatedly and reliably evokes a typical pattern of task-induced increase in heart rate and skin conductance level accompanied by typical respiratory changes over the course of the breathing exercise and recovery^22^. Therefore, we expected a comparable pattern of HV-induced heart rate, skin conductance, and respiratory changes in all groups upon repetition in both assessment sessions (i.e., prior to and after CBT^22^). In line with previous evidence, we expected a strong defensive response mobilization as indexed by a potentiation of the startle reflex after HV compared to a non-symptom-provocation control condition prior to CBT in all patients^9,22^. It has been demonstrated that repeated HV eliminates defensive reflex mobilization to HV-induced symptoms in persons that fear such symptoms and are at high risk to develop PD^22^. Therefore, and in accordance with previous evidence^17^, we assumed that the expected startle potentiation prior to CBT would no longer be present in patients who achieved significant clinical improvement following CBT. Moreover, we hypothesized that wait-list patients who did not undergo CBT and those who did not benefit from CBT would still exhibit a clear potentiation of the startle reflex, indicating a persistent activation of the defensive brain circuit to interoceptive threat.

## Materials and methods

### Participants

Fifty-eight treatment-seeking patients aged 18–56 years meeting DSM-IV diagnostic criteria for PD (with or without agoraphobia) were recruited from the outpatient clinic at the University of Greifswald. Diagnoses were determined using the Composite International Diagnostic Interview^34^ and verified by a certified psychotherapist. Thirty-eight patients were treated in accordance with a manualized protocol^7^ that was comprised of 12 weekly sessions of CBT focusing on therapist-guided interoceptive and in situ exposure exercises. Twenty patients were assigned to a wait-list control condition and treated after a 12-week delay (see supplement for detailed information). A pre (T1) and post (T2) treatment/waiting period characterization of the sample was realized using a combination of self-rating questionnaires and clinician-administered interviews. All subjects provided written informed consent prior to the study that was approved by the Ethics Committee of the German Society of Psychology and received financial compensation for their participation in the laboratory assessments. The study was registered on ClinicalTrials.gov (NCT04568109).

### Apparatus

Bioamplifiers (Coulbourn Instruments, Holliston, United States) registered electromyographic (EMG) activity over the left musculus *orbicularis oculi*, electrodermal and electrocardiographic activity, as well as respiration as described in the supplement. Data acquisition was realized using VPM software^35^.

### Materials, HV task, and procedure

To assess effects of psychotherapeutic treatment on defensive mobilization to an interoceptive threat, all participants underwent psychophysiological assessment at T1 and T2. An exemplary procedure of the psychophysiological assessment session is depicted in Fig. S1. After the signal quality was checked, each laboratory session started with a 2 min adaptation phase followed by a rating of the severity of the 14 DSM-IV panic symptoms on a Likert Scale ranging from 1 (not at all) to 10 (very strong) via computer keyboard (see supplement for further information). The interoceptive threat, that is, feared bodily sensations, were elicited using a highly standardized HV task (see supplement for further description). This HV procedure is highly efficient in inducing a variety of bodily sensations^20^ that persist for several minutes after the breathing exercise is discontinued^9^. Defensive mobilization was continuously assessed throughout the 10-min post-HV phase and retrospective ratings of HV-elicited symptoms were acquired as described above at the end of this phase. In addition to the described HV procedure, a 10-min control condition (no preceding symptom provocation) was introduced. During the post-HV and control phase, participants were instructed to sit comfortably while a black slide was presented. Each interoceptive threat/control phase was preceded by a colored slide (blue or yellow) indicating the upcoming HV/control condition. The order of control vs. HV phases was counterbalanced between subjects, i.e., half of the participants started with the HV condition and the other half with the control condition. The order of the control vs. HV phase was the same for T1 and T2.

A 50 ms burst of broadband white noise (95 dBA, rise/fall time <1 ms) was presented binaurally via AKG K-66 headphones (AKG Acoustics GmbH, Austria) to serve as a startle-eliciting stimulus. Presentation of startle probes was realized using VPM software^35^. During adaptation phase, eight startle probes were delivered to habituate startle response magnitudes to a stable baseline (mean inter-probe interval: 15 s; range: 10–20 s). Thirty startle probes (three per minute; mean inter-probe interval: 20 s; range: 10–30 s) were presented during both, the 10 min post-HV and the control phase, respectively. No startle probes were presented during the guided breathing task.

### Data reduction and analyses

The orbicularis oculi EMG was filtered, rectified, and smoothed offline. The onset (20–100 ms after probe delivery) and peak amplitude (within 150 ms after probe delivery) of the signal were manually determined. Startle response amplitudes (in µV) were standardized within each subject using *z*-score transformation and then transformed to *T*-scores ([*z* × 10] + 50; M = 50, SD = 10) to remove inter-individual variability not related to the experimental tasks. Skin conductance level, heart rate, petCO2, and respiratory rate were processed and derived from the recorded signals as described in the supplement.

To evaluate whether clinically significant improvement during CBT was associated with the elimination of dysfunctional defensive responding, in line with previous studies^7,17,36^, after completion of all clinical and psychophysiological assessments, patients of the CBT group were sub-divided based on therapy outcome data into a group that demonstrated clinically significant improvement and a group that did not show such an improvement. Clinical rating scales and self-report measures that have been used in previous clinical trials in PD patients as outcome measures and to determine treatment response were applied to define responders and non-responders in the current study^7,17,37–39^. Patients of the treatment group were classified as treatment responders if they achieved a *clinically significant change* (high-functioning end-state and a reliable change), i.e., if they scored 17 or less (i.e., none to mild anxiety severity) on the Hamilton Anxiety Scale (HAM-A) at post-assessment *and* showed statistically reliable change^40^ (see supplement for further information) in two or more of the following panic-specific outcome measures: number and severity of panic attacks, anxious apprehension, agoraphobic avoidance, and anxiety sensitivity (see also ref. ^36^ for studies using similar approaches to determine treatment responders). Patients of the treatment group not meeting these criteria were classified as non-responders, i.e., not fulfilling criteria for a clinically significant change (high-functioning end-state and a reliable change on two of four panic-specific outcomes). Panic-specific outcome measures used to determine treatment response were comprised of the anxiety sensitivity index^41^ as well as the anxious apprehension, panic attack, and agoraphobic avoidance subscales of the panic and agoraphobia scale^42^.

In all statistical analyses of physiological and self-report measures, a mixed-model analysis of variance (ANOVA) was applied. Baseline differences during the adaptation phase were evaluated including the between-subject factor group (wait-list controls vs. non-responder vs. responder) as well as session (T1 vs. T2) as a within-subject factor. Changes in respiration, heart rate, skin conductance level, and experienced symptoms from baseline levels to HV (manipulation check) were tested using onset (adaptation vs. first minute of HV) and session as within-subject factors. Moreover, the course of respiratory, skin conductance level and heart rate responding during HV was evaluated including minute (minutes 1 through 3) and session as within-subject factors. Respiratory, heart rate, and skin conductance level changes during the post-HV phase were examined including condition (post-HV vs. control phase), minute (minute 1 through 10), and session (T1 vs. T2) as within-subject factors. Moreover, the between-subject factors group and order (HV-control vs. control-HV) were included in these models. The effect of interoceptive threat on defensive reflex mobilization during the post-HV phase was examined including condition (interoceptive threat vs. control phase), minute (minute 1 through 10), and session (T1 vs. T2) as within-subject factors as well as group (wait-list controls vs. non-responder vs. responder) and order as between-subject factors. All statistical tests used a significance level of *p* < 0.05 (two-sided). Greenhouse–Geisser corrections of degrees of freedom were applied whenever necessary. For all significant *F*-tests, effect sizes (partial eta squared) are reported. Whenever assumptions necessary for conducting mixed-model ANOVA were violated, we also report nonparametric tests (Wilcoxon tests for within-subject repeated measures, Kruskal–Wallis–*H* tests for between-subject comparisons, or Friedman tests for within-subject repeated measures with more than two factor levels). All data were processed using SPSS 22.0 (SPSS for Windows, IBM). All data are depicted in figures as mean ± SEM. To illustrate a significant change in the potentiation of the startle response to the post-HV phase from T1 to T2, startle response magnitudes were averaged separately for the post-HV and control condition at T1 and T2 and difference scores were calculated by subtracting the mean startle responses during the control condition from the mean startle responses during the post-HV phase.

## Results

### Demographic and clinical characteristics

Demographic and clinical characteristics of patients at T1 and T2 assessment are summarized in Table 1. In line with previous studies using stringent criteria such as those used in the current study (i.e., multiple measures and modalities, reliable change, and clinical cutoff) to determine treatment response in CBT for PD^7,36^, in the present study 47.4% of patients of the treatment group were classified as responders, thus achieving a clinically significant improvement. As reported in Table 1, wait-list controls, treatment non-responder, and patients who showed a clinically significant improvement did not differ in sex, age, number of comorbid diagnoses, and self-reported or clinician-rated anxiety and panic symptomatology at T1, all *p*’s > 0.141. However, changes in clinical outcome measures from T1 to T2 differed between groups, Group × Session *p*’s < 0.032 (see Table 1). As expected, there was no change in clinical symptomatology in wait-list controls, all *p*’s > 0.058. There was a decrease in anxiety and panic symptomatology from T1 to T2 in the treatment group (see Table 1). This change was significantly stronger in patients who achieved clinically significant improvement as compared to patients classified as non-responders (except for MI-a and ACQ), by-group interactions (responder vs. non-responders) *p*’s < 0.043.

**Table 1:** Demographic and clinical characteristics of patients with PD at T1 and T2.

	Wait-list controls (*n* = 20)					Non-responder (*n* = 20)					Responder (*n* = 18)							
	T1		T2		T1 vs. T2	T1		T2		T1 vs. T2	T1		T2		T1 vs. T2	T1 group	T2 group	Group × Session
	Mean	SD	Mean	SD		Mean	SD	Mean	SD		Mean	SD	Mean	SD				
BDI (0–63)	15.8	9.1	14.6^a^	7.6	*p* = 0.242	13.9	9.0	12.2^a^	8.7	*p* = 0.425	15.8	12.0	6.1^b^	7.4	*p* = 0.004	*p* = 0.792	*p* = 0.007	*p* = 0.027
BSQ (17–85)	45.2	10.9	43.9^a^	13.4	*p* = 0.469	43.2	12.5	35.8^ab^	12.6	*p* = 0.017	47.0	14.5	27.3^b^	10.1	*p* < 0.001	*p* = 0.649	*p* = 0.001	*p* < 0.001
PAS (0–57)	22.0	9.0	17.9^a^	9.7	*p* = 0.143	18.2	9.7	11.8^ab^	8.3	*p* = 0.026	24.3	9.8	7.1^b^	7.6	*p* < 0.001	*p* = 0.142	*p* = 0.002	*p* = 0.001
Number/severity of panic attacks (0–4)	1.5	0.9	0.9	1.0	*p* = 0.059	1.4	1.0	1.3	1.2	*p* = 0.553	1.6	1.1	0.5	1.0	*p* < 0.001	*p* = 0.779	*p* = 0.101	*p* = 0.018
Agoraphobic avoidance (0–4)	1.8	1.3	1.4^a^	1.2	*p* = 0.114	1.5	1.3	0.7^b^	0.8	*p* = 0.005	2.0	0.9	0.4^b^	0.5	*p* < 0.001	*p* = 0.423	*p* = 0.002	*p* = 0.007
Anxious apprehension (0–4)	2.3	1.3	2.2^a^	0.9	*p* = 0.681	1.9	1.3	1.3^b^	1.0	*p* = 0.040	2.5	0.9	1.0^b^	0.8	*p* < 0.001	*p* = 0.235	*p* = 0.001	*p* = 0.005
ACQ (1–5)	2.0	0.5	2.0^a^	0.7	*p* = 0.469	2.0	0.6	1.5^ab^	0.3	*p* = 0.006	2.2	0.7	1.5^b^	0.6	*p* < 0.001	*p* = 0.652	*p* = 0.009	*p* = 0.010
MI alone (1–5)	2.2	0.9	2.3^a^	1.1	*p* = 0.124	2.4	1.2	1.7^ab^	0.8	*p* = 0.002	2.4	0.9	1.2^b^	0.4	*p* < 0.001	*p* = 0.842	*p* = 0.003	*p* = 0.006
ASI (0–64)	28.2	10.5	26.5^a^	13.3	*p* = 0.299	27.0	12.0	20.2^ab^	12.1	*p* = 0.016	34.0	13.6	13.6^b^	9.5	*p* < 0.001	*p* = 0.177	*p* = 0.008	*p* < 0.001
SIGH-A (0–56)	21.5	12.3	18.9^a^	13.2	*p* = 0.176	21.4	8.5	18.6^a^	8.4	*p* = 0.322	18.9	11.6	8.4^b^	5.3	*p* = 0.002	*p* = 0.715	*p* = 0.002	*p* = 0.024
CGI (1–7)	5.0	1.4	4.5^a^	1.0	*p* = 0.219	5.0	0.7	3.9^a^	1.2	*p* = 0.006	4.8	1.2	2.9^b^	0.9	*p* < 0.001	*p* = 0.835	*p* < 0.001	*p* = 0.031
Age	34.4	9.4				34.4	11.7				32.4	11.6				*p* = 0.820		
	*n*					*n*					*n*							
Sex (female/male)	13/7					11/9					11/7					*p* = 0.809		
Number of PD diagnoses with/without AG	19/1					17/3					18/0					*p* = 0.175		
Number of comorbid diagnoses (None/1/2–3)	10/6/4					9/8/3					8/6/4					*p* = 0.957		

*BDI* Beck Depression Inventory, *BSQ* Body Sensations Questionnaire, *PAS* Panic and Agoraphobia Scale. Number and severity of panic attacks: number of panic attacks during the last week as reported in the PAS. Agoraphobic avoidance as reported in the PAS. Anxious apprehension as reported in the PAS. *ACQ* Agoraphobic Cognitions Questionnaire, *MI alone* Mobility Inventory—alone subscale, *ASI* Anxiety Sensitivity Index, *SIGH-A* Structured Interview Guide for the Hamilton Anxiety Scale, *CGI* Clinical Global Impression Scale.

^a,b^Means with the same letter indicate that these groups did not differ significantly. Different letters indicate significant differences between groups.

^ab^Means were not significantly different from the means with the letters a and b.

### Adaptation phase

All physiological parameters and the number of reported panic symptoms during the adaptation phase did not differ between groups, *F*s < 2.41, *p*’s > 0.100, *Hs*(2) < 0.2.95, *p*’s > 0.228. Baseline SCL, p_et_CO_2_, and RR did not change from T1 to T2*, F*s < 2.64, *p*’s > 0.110, *Z*s > −1.50, *p*’s > 0.132, Session × Group *F*s < 1.74, *p*’s > 0.186. Baseline startle response magnitudes (in µV), heart rate and reported panic symptoms during the adaptation phase decreased from T1 to T2 in all groups, *F*s > 4.26, *p*’s < 0.045, $\eta _{\mathrm{p}}^2$ > 0.076, *Z*s < −2.10, *p*’s < 0.037, Session × Group *F*s < 1, *p*’s > 0.531.

### Symptom provocation: manipulation check

All patients included in the present analyses were fully compliant with the HV procedure.

#### Respiration

**Fig. 1:**
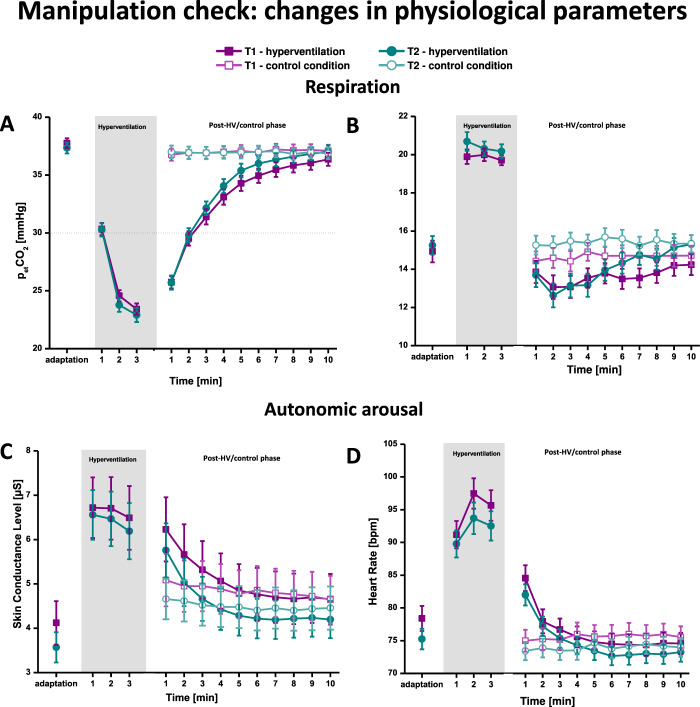
Manipulation check: changes in physiological parameters during adaptation, hyperventilation and the post-hyperventilation phase/control condition. Means of p_et_CO_2_ (**A**), respiratory rate (**B**), skin conductance level (**C**), and heart rate (**D**) during adaptation, hyperventilation as well as the post-hyperventilation interoceptive threat phase and control condition at baseline (T1) and post-treatment (T2).

As depicted in Fig. 1, during both assessment sessions, all patients reliably adjusted their respiratory rate to the pacing rhythm of 20 cycles/min, *F*(1, 46) = 66.18, *p* < 0.001, $\eta _{\mathrm{p}}^2$ = 0.606, *Z*(57) = −6.29, *p* < 0.001, Onset × Session *F*(1,46) = 1.09, *p* = 0.303, group or by-group interactions *F*s < 1.26, *p*’s > 0.296, group *H*(58) = 2.80, *p* = 0.247, and successfully maintained it throughout the HV task, *F*(2,82) = 1.35, *p* = 0.264, *χ*^2^(2) = 3.51, *p* = 0.172, Minute × Session *F*(2, 82) = 1.86, *p* = 0.174, group or by-group interactions *F*s < 2.07, *p*’ > 0.116, group *H*(58) = 1.40, *p* = 0.497. In all groups, HV led to the intended decrease in pCO2 towards the target pCO2 of 20 mmHg during T1 and T2, *F*(2, 98) = 189.95, *p* < 0.001, $\eta _{\mathrm{p}}^2$ = 0.795, *χ*^2^(2) = 82.79, *p* < 0.001, Minute × Session *F*(2,98) < 1, *p* = 0.424, group or by-group interactions *F*s < 2.58, *p*’s > 0.085, group *H*(58) = 1.90, *p* = 0.386; see S3 in the supplement). Thus, all patients dropped below the threshold of 30 mmHg that is critical for the elicitation body symptoms. After HV, the end-tidal pCO_2_ level continuously rose, converging towards non-challenge level towards the end of the 10-min post-HV phase (see Fig. 1 and Fig. S2 in the supplement), Condition × Minute *F*(9, 423) = 188.60, *p* < 0.001, $\eta _{\mathrm{p}}^2$ = 0.801. Respiratory rate was relatively decreased immediately after HV as compared to during the non-challenge control condition and recovered to non-challenge levels at the end of the post-HV phase, Condition × Minute *F*(9, 396) = 3.84, *p* = 0.009, $\eta _{\mathrm{p}}^2$ = 0.080. As expected, the observed respiratory pattern after HV was comparable in both sessions, Condition × Minute × Session, *F*s < 2.04, *p*’s > 0.083, and did not differ between groups, by-group interactions *F*s < 1.47, *p*’s > 0.192.

#### Heart rate and skin conductance level

In all groups, the onset of HV led to a strong initial increase in HR during both assessment sessions (see Fig. 1), *F*(1, 54) = 102.93, *p* < 0.001, $\eta _{\mathrm{p}}^2$ = 0.669, *Z*(58) = −6.51, *p* < 0.001, Onset × Session *F*(1,54) = 3.42, *p* = 0.070, $\eta _{\mathrm{p}}^2$ = 0.063, group or by-group interactions *F*s < 1.99, *p*’s > 0.147, group *H*(58) = 4.13, *p* = 0.127. During both assessment sessions, HR further increased until minute two of HV and then slightly decreased towards minute 3, *F*(2, 102) = 24.47, *p* < 0.001, $\eta _{\mathrm{p}}^2$ = 0.324, *χ*^2^(2) = 27.45, *p* < 0.001. This surge from minute 1 to 2 in HR was higher at T1 resulting in an increased HR during the last two minutes of HV at T1 compared to T2, Session × Minute *F*(2, 102) = 5.54, *p* = 0.008, $\eta _{\mathrm{p}}^2$ = 0.098. As expected, the onset of HV led to a pronounced increase in SCL at T1 and T2, *F*(1, 55) = 74.86, *p* < 0.001, $\eta _{\mathrm{p}}^2$ = 0.590, *Z*(58) = −6.32, *p* < 0.001, Onset × Session *F*(1, 55) < 1, *p* = 0.408, $\eta _{\mathrm{p}}^2$ = 0.013, group or by-group interactions *F*s < 1.80, *p*’s > 0.176, group *H*(58) = 1.43, *p* = 0.490. SCL slightly decreased throughout the HV task in both assessment sessions, *F*(2,102) = 4.64, *p* = 0.031, $\eta _{\mathrm{p}}^2$ = 0.082, *χ*^2^(2) = 28.17, *p* < 0.001, Minute × Session *F*(2,102) < 1, *p* = 0.469). As depicted in Fig. S3, there were no group differences in heart rate and skin conductance levels during HV, group or by-group interactions *F*s < 2.63 1, *p*’s > 0.054. In both laboratory sessions, HR and SCL was increased immediately after completion of the HV task and decreased to the level of the control condition within the first 2–3 min thereafter (see Fig. 1), Condition × Minute *F*s > 20.55, *p*’s < 0.001, $\eta _{\mathrm{p}}^2$ > 0.286, Condition × Minute x Session *F*s < 1.30, *p*’s > 0.264, $\eta _{\mathrm{p}}^2$ < 0.026. As expected, this observed pattern of heart rate and skin conductance level during post-HV was comparable in all groups (see Fig. S3), by-group interactions *F*s < 1.36, *p*’s > 0.247. (There was a significant Condition × Minute × Session × Group interaction in SCL, *F*(18, 459) = 3.90, *p* = 0.005. Post hoc test revealed that, in non-responder, SCL after HV decreased to the level of the control condition more quickly during T2 compared to T1, Condition × Minute × Session *F*(9, 162) = 4.51, *p* = 0.018; Condition × Minute × Session *F*s 2.66, *p*’s > 0.094 for wait-list controls and responders.)

#### Symptom reports

**Fig. 2:**
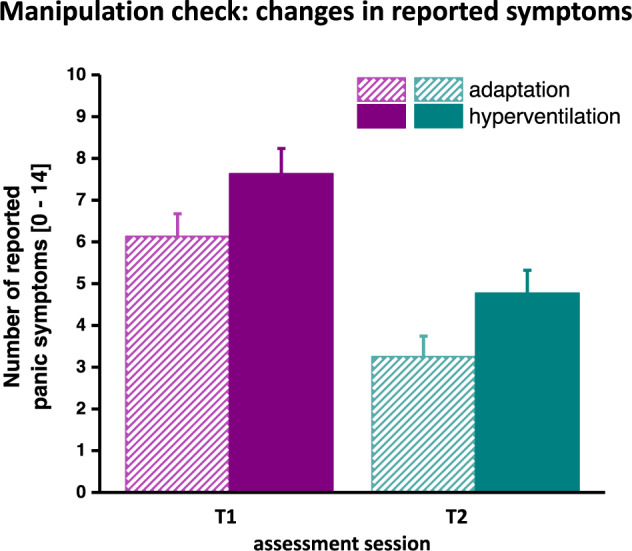
Manipulation check: changes in reported panic symptoms from the adaptation phase to the hyperventilation phase. Means and standard errors of the number of reported panic symptoms during adaptation and hyperventilation at T1 and T2, respectively.

In both assessment sessions, all groups reported more panic symptoms during HV compared to during adaptation, *F*(1,55) = 19.14, *p* < 0.001, $\eta _{\mathrm{p}}^2$ = 0.269, *Z*(58) = −5.11, *p* < 0.001, Onset × Session *F*(1, 55) = 1.09, *p* = 0.914, group or by-group interactions *F*s < 1.90, *p*’s > 0.173. As depicted in Fig. S4, palpitations, sweating, paresthesia, dyspnea, fear of losing control, and fear of dying were the most frequently reported symptoms during HV. As seen in Figs. 2 and S4, the overall number of reported panic symptoms decreased from T1 to T2, *F*(2, 55) = 30.49, *p* < 0.001, $\eta _{\mathrm{p}}^2$ = 0.370, *Z*(58) = −4.20, *p* < 0.001. This decrease did not differ between groups (see Fig. 2), *F*(2, 55) = 1.97, *p* = 0.149.

### Defensive reflex mobilization

**Fig. 3:**
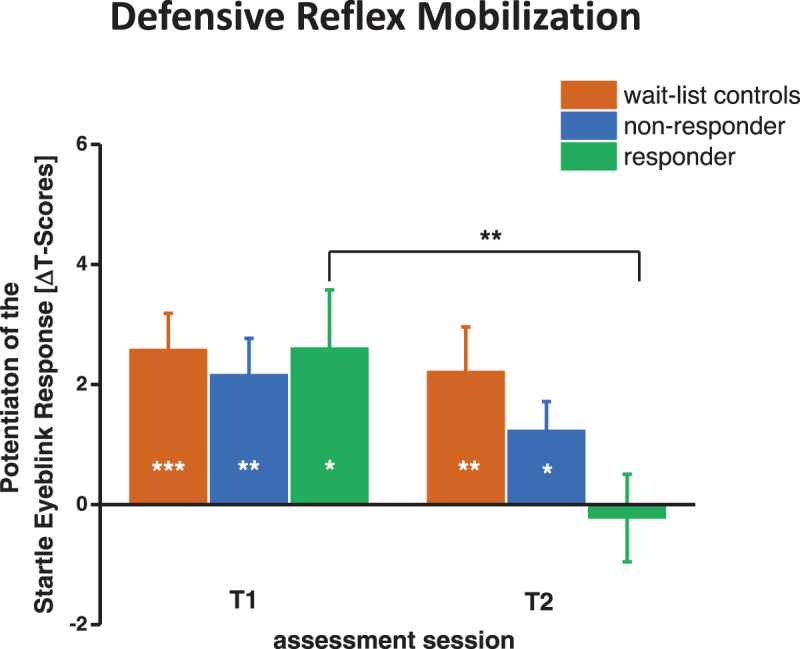
Defensive reflex mobilization during the post-hyperventilation phase vs. control condition. Means and standard errors of the potentiation of the startle eyeblink reflex during the post-hyperventilation phase vs. control condition in wait-list-controls, treatment non-responder and responder at T1 and T2. Asterisks denote a significant potentiation of the startle eyeblink response during the post-HV phase compared to the safe condition. In responders, asterisks indicate a significant reduction of this potentiation from T1 to T2. ***p < 0.001, **p < 0.01, *p < 0.05.

As depicted in Fig. 3, during T1, patients showed a pronounced potentiation of startle response magnitudes during the post-HV interoceptive threat phase as compared to during the control condition, *F*(1, 50) = 32.45, *p* < 0.001, $\eta _{\mathrm{p}}^2$ = 0.394, *Z*(58) = −5.21, *p* < 0.001. This potentiation of the startle response magnitudes did not differ between patient groups, *F*(2, 50) < 1, *p* = 0.723, *H*(58) = 0.989, *p* = 0.989. Most importantly, from T1 to T2, the potentiation of the startle eyeblink response significantly decreased only in responders, *F*(1, 15) = 11.41, *p* = 0.004, $\eta _{\mathrm{p}}^2$ = 0.432, *Z*(18) = −2.55, *p* = 0.011, but did not significantly change in non-responders and wait-list controls, *F*s < 1.22, *p*’s > 0.287, $\eta _{\mathrm{p}}^2$ < 0.068, *Zs* > −1.60, *p*’s > 0.108, Session × Condition × Group *F*(2, 50) = 3.27, *p* = 0.046, $\eta _{\mathrm{p}}^2$ = 0.116. However, only in those patients who showed clinically significant improvement (responder), startle response magnitudes were no longer potentiated during confrontation with interoceptive threat after CBT, *F*(1, 15) < 1, *p* = 0.750, *Z*(18) = −0.02, *p* = 0.983. As depicted in Fig. 3, the wait-list control group and non-responders continued to exhibit a significant potentiation of the startle eyeblink response, *F*s > 6.01, *p*’s < 0.026, $\eta _{\mathrm{p}}^2$ > 0.261, *Z* > −2.23, *p*’s < 0.026, Condition × Group *F*(2, 50) = 3.81, *p* = 0.029, $\eta _{\mathrm{p}}^2$ = 0.132. Importantly, as can be seen in Fig. S5 (supplement), groups did not differ significantly in startle response magnitudes in the control condition during T1 and T2, control condition: session (T1 vs. T2) *F*(1, 50) = 1.435, *p* = 0.237, *Z*(58) = −1.15, *p* = 0.250, Session × Group *F*(1, 50) < 1, *p* = 0.752, indicating that startle potentiation in wait-list controls and treatment non-responders after treatment did not result from a reduction of startle response magnitudes during the control condition from T1 to T2.

## Discussion

The present study documents effects of CBT on defensive activation to feared body symptoms in patients with PD. As expected, at T1, all patients showed a strong defensive response mobilization as indexed by a potentiation of the amygdala-dependent low-level brain stem startle reflex during exposure to interoceptive threat compared to a control condition. Importantly, patients who achieved clinically significant symptom improvement and those who did not show such an improvement during CBT as well as patients of the wait-list control condition did not differ in panic and anxiety symptomatology at T1. After CBT, patients who showed a clinically significant improvement as indexed by reported panic symptomatology no longer exhibited a defensive response mobilization to interoceptive threat. In contrast, treatment non-responders and wait-list controls continued to show a clear defensive response mobilization to feared body symptoms at T2. Importantly, all patients adhered to the HV task. As expected, and in line with previous studies that also applied a well-controlled HV task^22^, in the current study, HV induced increases in heart rate and skin conductance level as well as a variety of bodily symptoms that was comparable across participants, and during both lab assessments, suggesting that symptom provocation was similar in all patients.

Previous evidence demonstrated that in comparison to low fearful control individuals, persons who fear body symptoms, show an increased defensive response mobilization during exposure to feared body symptoms^9,10,22^. In the present study, at T1, we observed a pronounced defensive response mobilization as indexed by a potentiation of the startle reflex elicited in the presence of HV induced body symptoms in patients with PD. Evidence from animal and human research revealed that the potentiation of this brain stem reflex is mediated by brain defensive networks with the amygdala being the central hub^23–25^. Previous neuroimaging studies in PD patients demonstrated a dysfunctional activation in this brain network^15,17–19^, e.g., an exaggerated defensive activation to actually innocuous stimuli like conditioned safety stimuli, that have been presented in a context of external threat. The present study extends this evidence in demonstrating defensive activation to actually innocuous *interoceptive* stimuli in patients with PD as compared to non-anxious healthy controls who showed no indication of defensive activation to HV-induced symptoms in previous studies^9,22^. This is consistent with contemporary learning accounts of PD^6^ proposing that, via associative learning processes, actually innocuous body sensations may become interoceptive threat signals thus activating these defensive networks.

Most importantly, the present data indicate that this defensive activation to feared body symptoms was eliminated immediately after successful exposure-based therapy. After treatment, we found no potentiation of the startle reflex to HV induced body symptoms in patients who showed a clinically significant improvement, suggesting that interoceptive body sensations no longer activate defensive responses in this patient group. In contrast, in wait-list controls, defensive activation to body symptoms did not change when retested after a waiting period matched to the treatment duration. Interestingly, it has previously been demonstrated that individuals with low trait fear of body symptoms also exhibited no potentiation of the startle response to exposure to body symptoms^9,22^, supporting the view that treatment responders show a more functional response to body symptoms after treatment. Accordingly, neuroimaging data revealed that clinical improvement during CBT is linked to a normalization of neurofunctional activation ensuring a functional down-regulation of defensive activation^15–17,19^ that might account for the normalization in defensive response mobilization to feared body symptoms in treatment responders in the present study.

In contrast to treatment responders, patients who did not show clinically significant improvement after CBT still demonstrated a defensive mobilization to HV-elicited body symptoms, albeit the intensity was slightly reduced. Thus, persistent anxiety and panic symptomatology as shown in wait-list controls and non-responders are accompanied by defensive mobilization to body sensations. The present finding corroborates neuroimaging studies that reported an impaired down-regulation of the activation of defensive circuits in treatment non-responders^17^ that might be responsible for the persistent defensive mobilization when experiencing mild body symptoms. In accordance with models of the development and maintenance of PD^6^, one can assume that this defensive response mobilization to body symptoms might increase the risk to experience new panic attacks that increase anxious apprehension as well as to show excessive avoidance, thus contributing to the chronicity of anxiety and panic symptomatology and functional impairment. Thus, the present evidence in treatment non-responders calls for the implementation of optimized exposure strategies (see ref. ^43^ for detailed information on optimized exposure in general or ref. ^44^ for the optimization of interoceptive exposure) to facilitate extinction of threat associations (i.e., body sensations indicating threat) to decrease the risk that is associated with persistent excessive defensive mobilization to body symptoms, i.e., the chronicity of psychopathological symptoms.

Several limitations of the present findings need to be commented on. It is possible that treatment effects in non-responders were not detected due to the relatively small sample size. Although non-responders showed a slight decrease in startle potentiation from pre to post CBT, startle reflex mobilization after CBT remained present. Possibly, treatment non-responders in the present study did not show a sufficient decrease in clinical symptomatology that would be reflected in a more functional response to body symptoms as observed in responders. Notwithstanding the above, further studies with larger samples are warranted to replicate the findings of the present study. Moreover, in the present study, the presence of comorbid depressive and anxiety disorders in our sample of patients with a principal diagnosis of PD might affect treatment effects on defensive activation to body symptoms. However, the characteristics of the present sample correspond to previous treatment and epidemiological studies^7,45^ and comply with the clinical picture of patients with PD observed in clinical practice raising the external validity of the study. Importantly, the number of comorbid diagnoses did not differ between responders, non-responders, and wait-list controls. Most importantly, there were no differences in depressive symptomatology between groups, indicating that the impact of depressive symptomatology on the present results was equal across groups. Moreover, psychotropic medication was allowed. The results of the current study might indicate that psychotropic medication had no impact on the observed effects of CBT on defensive responding (see supplement). However, it is possible that the effects of psychotropic medication on defensive responding were not detected due to the relatively small and unequal sample sizes. In the present study, the response rate to CBT was relatively low. However, comparable response rates were found in previous studies applying stringent criteria to determine treatment response^36^. Moreover, it is to note that patients who were classified as treatment responder in the present study not only improved but also achieved a high functioning end-state following CBT. Moreover, the use of a wait-list control group in the present study does not allow to delineate the effects of treatment engagement or nonspecific treatment factors on clinical and psychophysiological outcomes. Future studies ought to implement an active control condition to control for nonspecific treatment factors and treatment engagement.

## Conclusions

In the present study, a symptom provocation challenge was used to examine effects of CBT on defensive mobilization to actually harmless body sensations in patients with PD. It was demonstrated that treatment response was accompanied by changes in defensive activation to feared body symptoms. The present study indicates that a normalization of defensive activation to body symptoms is accompanied by clinically significant improvement in patients with PD, while persistent defensive mobilization is associated with persistent psychopathological symptoms and functional impairment. In line with studies that have coupled defensive reflex mobilization to treatment outcome in fear and trauma-related disorders^14,30,31^, the present study suggests that the elimination of startle potentiation to interoceptive threat might serve as a neurobiological correlate of treatment response in PD. Therefore, the neuroscience-based approach used in the present study might be a useful tool for the transdiagnostic characterization of dysfunctions in neurobiological systems and their behavioral outcome in anxiety disorders, including their changes during treatment beyond symptom reports. In this regard, future studies should include modern neuroimaging techniques to reveal changes in neurofunctional activation in defensive brain circuits that might be linked to the elimination of startle response potentiation to interoceptive threat after successful CBT. The present findings enhance our knowledge on neurobiological correlates of treatment response and may help to monitor and develop optimized and individualized treatment to maximize the effectiveness of exposure-based therapies.

## Supplementary information

SUPPLEMENTAL MATERIAL
