# Supplementary material for: Decreased defensive reactivity to interoceptive threat after successful exposure-based psychotherapy in patients with panic disorder
Source: Transl Psychiatry. 2021 Mar 17;11:177. doi: 10.1038/s41398-021-01298-7 (PMC7969920; doi:10.1038/s41398-021-01298-7)
Supplement: Supplementary file 1 — SUPPLEMENTAL MATERIAL [file 41398_2021_1298_MOESM1_ESM.docx]

**Supplementary materials**

**Decreased defensive reactivity to interoceptive threat after successful exposure-based psychotherapy in patients with panic disorder**

Christoph Benke, Manuela G. Alius, Alfons O. Hamm, & Christiane A. Pané-Farré,

**Supplementary methods**

**Participants**

Exclusion criteria were current suicidal intent, any psychotic or bipolar disorder, borderline personality disorder, a medical condition that could explain patients’ symptoms, or physical contradictions regarding application of exposure-based CBT (e.g., neurological disease). PD (with or without agoraphobia) had to be the principal diagnosis which was verified by a certified psychotherapist. Presence of comorbid diagnoses (e.g., other anxiety disorders) was allowed. Physical health-related medication and psychotropic medication (e.g., SSRI, or SNRI) except for benzodiazepines was allowed. Patients had to be on a stable psychopharmacological medication schedule prior to study entry for at least 12 weeks and were encouraged to maintain medication constant throughout the treatment and both assessment sessions. Groups significantly differed in psychotropic medication status: 50 % of wait-list controls (*n* = 10), 45 % of non-responders (*n* = 9), and 0 % of responders (*n* = 0) were currently treated with psychotropic medication, *χ*^2^(2)=12.83, *p*=.002. However, as reported in Table S1, groups did not differ significantly regarding medication with specific classes of drugs (e.g., SSRIs).

The sample size calculation was based on effects reported in a study using repeated hyperventilation in high anxiety sensitive individuals that parallel patients with PD in their fear of body symptoms^1^. A sample size of 17 subjects per group was calculated to detect a large effect ($\eta^{2}$ = .26) and to achieve a power of 95% for a mixed model ANOVA including a within-subject factor and a between-subjects factor as well as a within-between-subjects interaction. In line with previous studies, we expected a treatment response rate of 45%-50%^2^. Therefore, we aimed to investigate between 51 and 60 patients in total to achieve a sample size of 17-20 patients per group.

**Table S1** *Frequency of specific classes of drugs in wait-list controls, non-responder, and responder*

| Specific drug classes | wait-list  controls | | |  | non-responder | |  | | responder | | | |  | | statistics | | |
| --- | --- | --- | --- | --- | --- | --- | --- | --- | --- | --- | --- | --- | --- | --- | --- | --- | --- |
|  | % | *n* |  | | % | *n* | |  | % | *n* |  | | *χ*^2^ | | | | *p* |
| SSRIs | 26.3 | 5 |  | | 15.8 | 3 | |  | 0 | 0 | |  | | 5.02 | | .081 | |
| SNRIs | 5.3 | 1 |  | | 10.5 | 2 | |  | 0 | 0 | |  | | 1.93 | | .380 | |
| Tri-/tetracyclic  anti-depressants | 21.1 | 4 |  | | 15.8 | 3 | |  | 0 | 0 | |  | | 5.02 | | .081 | |
| Atypical antipsychotics | 0 | 0 |  | | 5.3 | 1 | |  | 0 | 0 | |  | | 1.93 | | .380 | |

*Note.* SSRIs: selective serotonin reuptake inhibitors; SNRIs: serotonin-norepinephrine reuptake inhibitors

**Treatment**

The exposure-based CBT group was treated in accordance with a manualized protocol^3^ that was comprised of 12 weekly sessions of exposure-based CBT. Sessions 1–3 consisted of psychoeducation and an individualized behavioral analysis of the patient’s symptoms and coping behaviors (e.g., avoidance behavior). In sessions 4–5 the treatment rationale for exposure (i.e., sufficiently long and repeated exposure to feared stimuli without utilization of safety behaviors leads to fear reduction) was introduced and interoceptive exposure exercises were implemented during the therapy session. Sessions 6–8 were comprised of three standardized in situ exposure exercises (bus, shopping mall, and forest) which were accompanied by the therapist. During exposure, therapists provided feedback, modeled correct implementation, monitored anxiety levels, and corrected any use of safety behaviors. Exposure in situ was enriched by interoceptive exercises. Homework was implemented to practice the exposure and engage in self-monitoring of the exposure in situ exercises. Patients were instructed to complete two exposure in situ exercises in addition to the exposure session accompanied by the therapist. Session 9 reviewed progress to date and addressed changes in anticipatory anxiety. In sessions 10–11 the patients’ individual two most significant feared situations were selected for in situ exposures. Session 12 rehearsed crucial exposure related elements of the manual, reviewed treatment progress, anticipated difficulties, strategized solutions for the patients’ feared situations and avoidance behavior, and instructed patients to continue exposing themselves to feared situations.

**Wait-list control condition**

Participants of the wait-list control condition completed the T1 assessment session as well as the T2 assessment session after a 12-week waiting period. They received no treatment between T1 and T2. After completion of the T2 assessment, all participants of the waitlist control condition were treated with the manualized CBT protocol mentioned above.

**Materials, Apparatus and hyperventilation task**

The hyperventilation task was introduced to the participants as a “fast breathing exercise”. During the 3 min HV task, tones of rising and falling pitch were presented via headphones prompting the participants to breathe at a respiratory rate of 20 cycles/min. To assess compliance with the HV procedure, the respiratory rate (RR) and the CO2 of the expired air (p_et_CO2) were continuously monitored by a Nellcor NPB-70 Capnograph (Nellcor Puritan Bennett, Pleasanton, CA). Visual feedback (instruction slides) was used to lead the participant to ‘‘breathe deeper’’ until a target p_et_CO2 of 20 mmHg was reached. Using further visual feedback (‘‘breathe more shallow’’, ‘‘deeper,’’ or at a ‘‘constant depth’’), the breathing depth was adjusted throughout the hyperventilation task to maintain the target p_et_CO_2_. The visual feedback was given by the experimenter who tracked p_et_CO_2_ levels online.

To measure the eyeblink component of the startle response the EMG activity was recorded by VPM software using two electrolyte-filled (Marquette Hellige, Freiburg, Germany) Ag/AgCl miniature surface electrodes (4 mm diameter, Sensormedic, Yorba Linda, CA) attached over the orbicularis oculi muscle beneath the lower left eyelid. Skin impedance was not measured. The raw EMG signal was amplified by a Coulbourn S75-01 amplifier and filtered with a 30 Hz high-pass and a Kemo KEM-VBF8-03 400 Hz low-pass filter. Digital sampling was carried out at a rate of 1000 Hz via a 12-bit A/D converter starting 100 ms before the onset of the startle stimulus and lasting 400 ms after probe onset.

Skin conductance was recorded from the hypothenar eminence on the palm of the participants' non-dominant hand using two Ag/AgCl standard electrodes (8 mm diameter, Marquette Hellige) filled with a 0.05 M sodium chloride electrolyte medium. A constant DC voltage of 0.5 V was applied across electrodes (attached 15 mm apart) by a Coulbourn S71-22 skin conductance coupler that processed the signal with a resolution of 0.01 µS. The DC voltage amplified signal was continuously sampled at 10 Hz by a 12-bit A/D-converter.

Electrocardiogram (ECG) was measured with electrolyte filled Ag/AgCl standard electrodes (Marquette Hellige) placed in an Einthoven-II-setup. The raw ECG signal was amplified and filtered through a 0.1 - 13 Hz band-pass filter using a Coulbourn S75-01 bioamplifier. The digital sampling rate was set to 100 Hz.

Respiratory parameters were registered by an inductive plethysmography system (Respitrace, Q.D.C., SensorMedics, NewMedics GmbH, Öhringen, Germany) applying thoracic and abdominal respiration belts.

End-tidal carbon dioxide partial pressures (p_et_CO_2_) were registered by a Nellcor NPB-70 capnograph analyzing the amount of pCO_2_ present at the end of exhalation (p_et_CO_2_) via infrared spectroscopy. For this purpose, nasal prongs were placed in both nostrils from which the expired air was continuously drawn and delivered through a 1.2 mm diameter tube to the monitor. All respiratory outputs were continuously digitized with a sampling rate of 10 Hz.

In the present study, participants were asked to rate the severity of 14 panic symptoms listed in the DSM IV on a Likert Scale ranging from 1 (not at all) to 10 (very strong) via computer keyboard (e.g., “During hyperventilation, how severely have you had … sensations of shortness of breath or dyspnea”). The original DSM IV panic symptom “feeling dizzy, unsteady, light-headed, or faint” was split-up in two separate items: “feeling unsteady or dizzy” and “feeling faint”. Thus, in the present study, participants rated the following 14 panic symptoms: palpitation or accelerated heart rate; sweating; trembling or shaking; sensations of shortness of breath or dyspnea; feeling of choking; chest pain or discomfort; nausea or abdominal distress; feeling unsteady or dizzy; feeling faint; feelings of unreality or being detached from oneself; fear of losing control or “going crazy”; fear of dying; numbness or tingling sensation; chills or hot flushes .. Rating options were projected onto a 1.50 x 1.30 m screen in front of the participants. Presentation of ratings was realized using Presentation software (Neurobehavioral Systems, Inc.).

**Data reduction and analysis**

The raw EMG signal was filtered off-line with a 60 Hz high-pass filter to remove eye movement artifacts and then rectified and smoothed using a first-order low-pass filter with a time constant of 10 ms. Then, the startle eyeblinks were semi-automatically scored using a computer program ^4^ that identified blink onset (20-100 ms after probe delivery) and peak amplitude (within 150 ms after probe delivery). Each individual startle eyeblink response was checked and manually corrected when necessary. Only trials in which blinks started during 20-100 ms after delivery of the startle probe and reached their peak amplitude within 150 ms were scored as valid startle responses. If no blink was detected in the defined time window, the trials were scored as zero responses. In correspondence with the guidelines for human startle eyeblink studies^5^, trials were rejected (1.7 %) and treated as missing values if there was excessive noise due to technical problems, spontaneous eye-blinks, or movement artifacts. Digital values were converted to µV and then exported. To remove inter-individual variability not related to the experimental manipulation, all values were transformed to T-scores (*M* = 50, *SD* = 10) as recommended by the guidelines for human startle eyeblink studies^5^. Raw startle response magnitudes in µV were used to evaluate baseline differences between groups.

Digital values of skin conductance level (SCL) were converted to µS and exported in 30-s means.

The ECG signal was visually inspected, movement artifacts set to missing, and misplaced R-wave triggers were corrected using ANSLAB version 2.4 (Autonomic Nervous System Laboratory, University of Basel, Switzerland). Inter-beat-intervals were calculated, converted to heart rate (HR in bpm), and then averaged across 30s-bins.

Values of end-tidal pCO_2_ were exported in 10-s means. To enable a calculation of volume data for the entire session a regression coefficient was calculated which expresses the association between the digital outputs of the spirometry (volume) and Respitrace system (band stretch). For this purpose, the Respitrace sum channel and the spirometry outputs were visually checked using ANSLAB version 2.4 (Autonomic Nervous System Laboratory, University of Basel, Switzerland). Technical (resets of the system) and body movement artifacts were removed and treated as missing values. Finally, respiratory rate (RR; in bpm) was calculated for each breath and exported in 30-s means.

To determine the reliable change in panic-specific outcome measures (i.e., number and severity of panic attacks, anxious apprehension, agoraphobic avoidance, and anxiety sensitivity), we apply the Jacobson & Truax^6^ reliable change index in which the reliable change is determined using the following formula: reliable change = (post-treatment score – pre-treatment score)/standard error of the difference. The standard error of differences was computed according to the formula suggested by Jacobson & Truax^6^: standard error of differences = square root of (square root of 2*((variance of measure at pretreatment)*(1-measure reliability))2). The average Cronbach alpha across pre and post was used as the reliability measure. A reliable change index greater than 1.96 indicate that changes in outcomes from pre to post are statistically reliable (p < .05). In the present study, patients of the treatment group that achieved a reliable change (reliable change index greater than 1.96) in two or more panic-specific outcome measures (number and severity of panic attacks, anxious apprehension, agoraphobic avoidance, and anxiety sensitivity) and scored 17 or less (i.e., none to mild anxiety severity) on the Hamilton Anxiety Scale (HAM-A) at post-assessment were classified as responders who achieved a clinically significant change (high-functioning end-state and a reliable change).For explorative analyses of effects of psychotropic medication on psychophysiological and subjectively reported symptoms, the additional between-subject factor drug (psychotropic medication vs. no psychotropic medication) was included in analyses of the adaptation, HV and post-HV phase.

**Supplementary Figures**


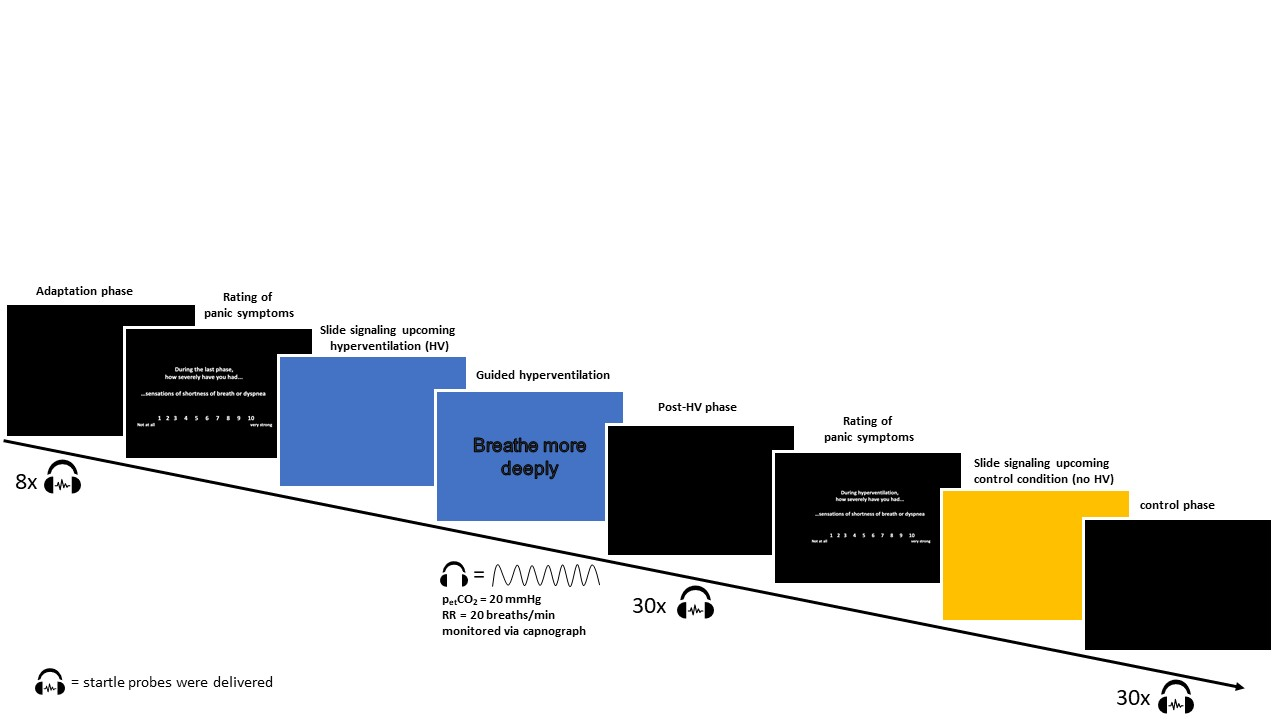


**Fig. S1** Description of procedures for the psychophysiological assessment session at T1 and T2 (i.e., prior to and after a 12-week CBT or waiting period). Each experiment started with a 2 min adaptation phase followed by ratings of panic symptoms. Then, one half of the participants started with the hyperventilation (HV) condition, which was comprised of a 3-min guided HV and 10 min post-HV phase followed by ratings of panic symptoms, while the other half started with the control condition, which was comprised of a 10-min control phase that was not preceded by a HV phase. A colored slide (yellow or blue) indicated the upcoming HV/control condition. During HV, tones of rising and falling pitch were presented via headphones to prompt the participants to breath at a rate of 20 breaths per minute. Visual feedback (‘‘breathe more shallow’’, ‘‘deeper,’’ or at a ‘‘constant depth’’) was presented on the screen to lead the participants to achieve and maintain a target p_et_CO_2_ level of 20 mmHg. The visual feedback was given by the experimenter who tracked p_et_CO_2_ levels online.


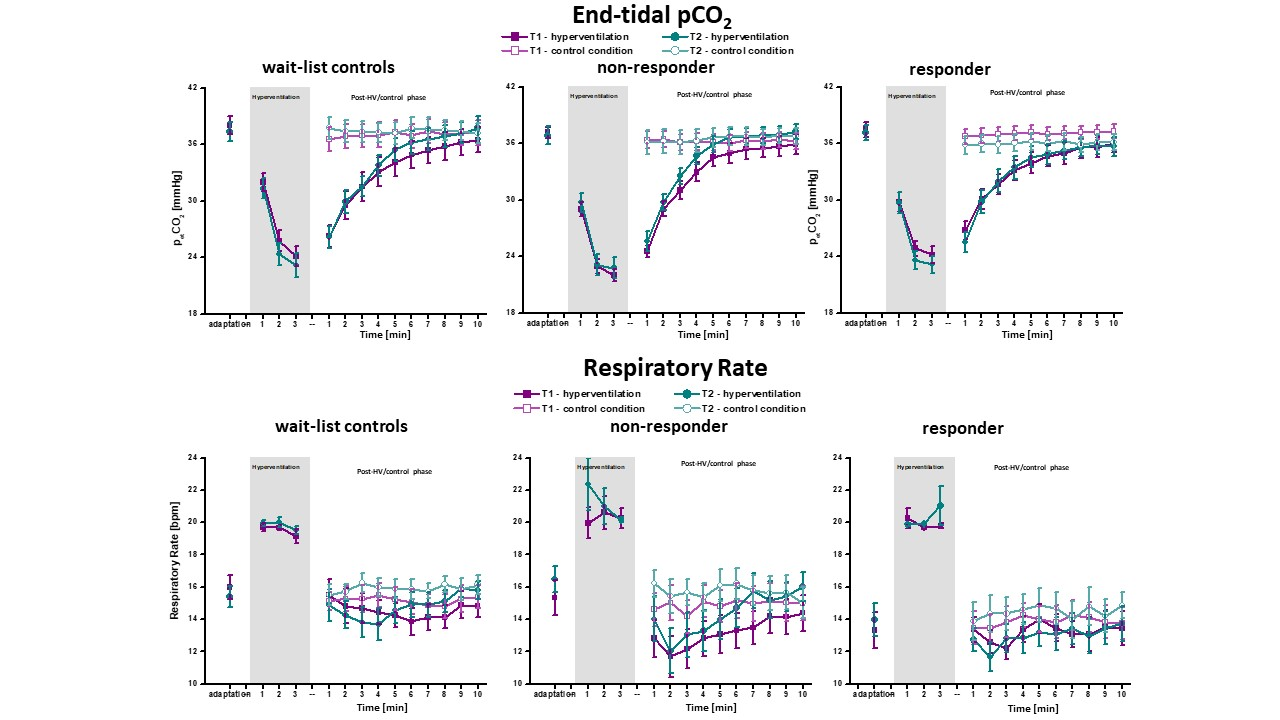


**Fig. S2** Means of p_et_CO_2_ (upper panel) and respiratory rate (lower panel) during adaptation, hyperventilation as well as the post-hyperventilation interoceptive threat phase and control condition at T1 and T2 in wait-list controls (left), non-responder (middle) and responder (right).


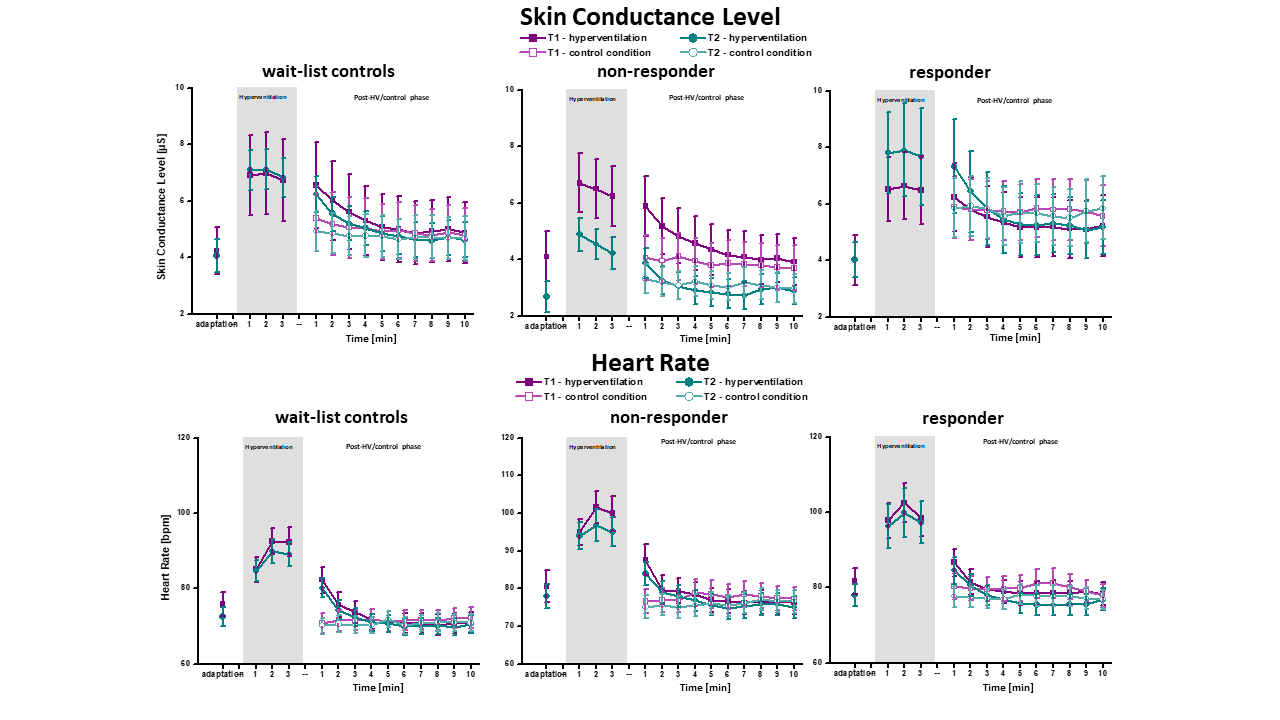


**Fig. S3** Means of skin conductance level (upper panel) and heart rate (lower panel) during adaptation, hyperventilation as well as the post-hyperventilation interoceptive threat phase and control condition at T1 and T2 in wait-list controls (left), non-responder (middle) and responder (right).

**Fig. S4** Frequency of reported panic symptoms experienced during HV at T1 and T2.


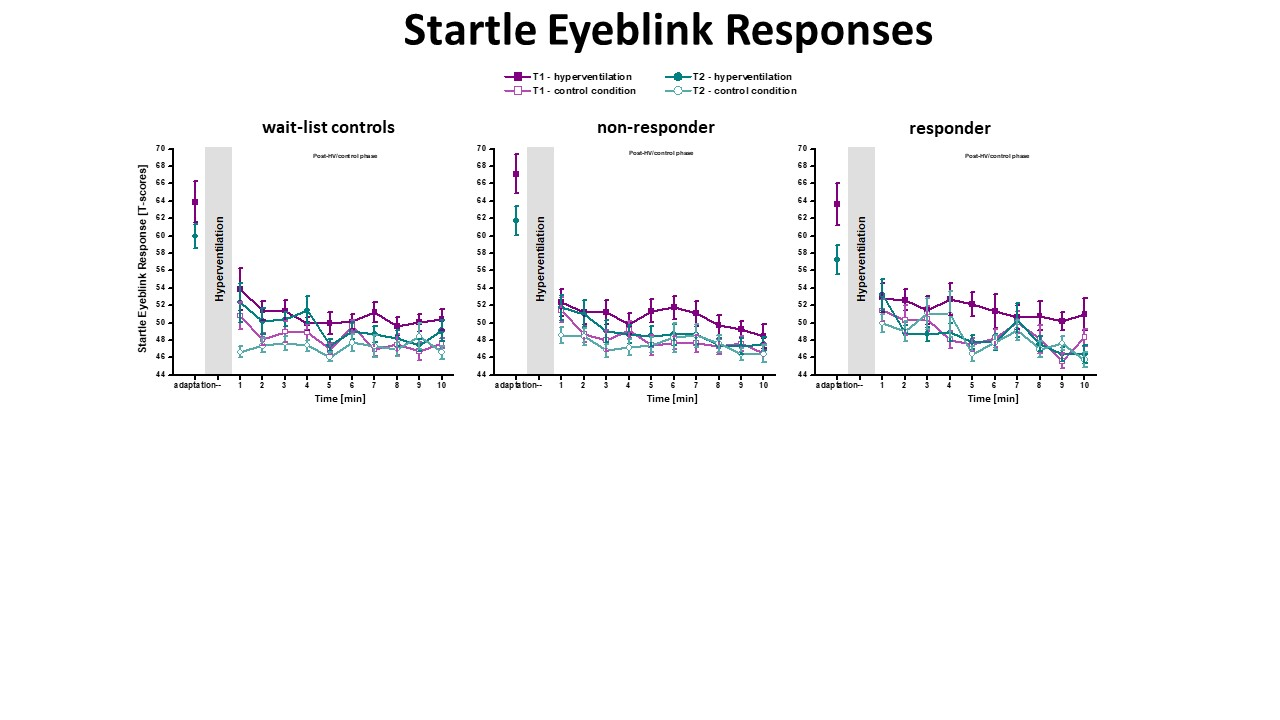


**Fig. S5** Means of startle eyeblink responses during adaptation and the post-hyperventilation interoceptive threat phase and control condition at T1 and T2 in wait-list controls (left), non-responder (middle) and responder (right).

**Effect of psychopharmacological treatment on physiological and subjective reactivity**

***Effects of psychopharmacological treatment on baseline reactivity***

There was no effect of psychotropic medication on baseline HR, SCL, p_et_CO_2_, RR, startle response magnitudes and reported panic symptoms at T1 and T2, all drug and group by drug interactions, *F*s<2.87, *p*s>.096.

***Symptom provocation: manipulation check***

The observed pattern of heart rate and skin conductance level during HV was not modulated by psychotropic medication, *F*s<3.38, *p*s>.056, $\eta_{p}^{2}$<.089, except that, in HR, only persons who took psychotropic medication showed the observed decrease from minute 2 to minute 3, *F*(1, 16)=5.30, *p*=.035, while those persons who took no psychotropic medication did not show this decrease in HR, *F*(1, 36)=2.12, *p*=.154, Time x Drug *F*(2, 104)=3.97, *p*=.038, $\eta_{p}^{2}$=.036. The observed pattern of respiration during HV was not modulated by psychotropic medication, all drug and by-drug interactions *F*s < 2.06, *p*s > .158, $\eta_{p}^{2}$< .048. Psychotropic medication had no effect on the observed increase in reported panic symptoms from the adaptation phase to HV, all interactions including onset and drug *F*s < 1.10, *p*s > .300, $\eta_{p}^{2}$< .021. The observed pattern of respiration, heart rate and skin conductance level after HV compared to the control condition was not affected by psychotropic medication, all interactions including condition and drug *F*s < 3.87, *p*s > .055, $\eta_{p}^{2}$< .080.

***Defensive reflex mobilization***

Psychotropic medication did not affect the modulation of the startle eyeblink response during the post-HV interoceptive threat phase vs. control condition, all interactions including condition and drug *F*s < 1.57, *p*s > .219, $\eta_{p}^{2}$< .031.

**References**

1. Holtz K., Hamm A.O. & Pané-Farré C.A. Repeated Interoceptive Exposure in Individuals With High and Low Anxiety Sensitivity. *Behav Modif* **39**, 014544551877226 (2018).

2. Loerinc A.G., Meuret A.E., Twohig M.P., Rosenfield D., Bluett E.J. & Craske M.G. Response rates for CBT for anxiety disorders: Need for standardized criteria. *Clin Psychol Rev* **42**, 72–82 (2015).

3. Gloster A.T. *et al.* Psychological treatment for panic disorder with agoraphobia: A randomized controlled trial to examine the role of therapist-guided exposure in situ in CBT. *Journal of Consulting and Clinical Psychology* **79**, 406–420 (2011).

4. Globisch J., Hamm A.O., Schneider R. & Vaitl D. A computer program for scoring reflex eyeblink and electrodermal responses written in PASCAL. *Psychophysiology* **30**, S30 (1993).

5. Blumenthal T.D., Cuthbert B.N., Filion D.L., Hackley S., Lipp O.V. & van Boxtel A. Committee report: Guidelines for human startle eyeblink electromyographic studies. *Psychophysiology* **42**, 1–15 (2005).

6. Jacobson N.S. & Truax P. Clinical significance: A statistical approach to defining meaningful change in psychotherapy research. *Journal of Consulting and Clinical Psychology* **59**, 12–19 (1991).
